# Frequency of Multivessel Severe Coronary Artery Disease in Patients With Non-ST Segment Elevation Myocardial Infarction Having Markedly Raised Cardiac Troponin T

**DOI:** 10.7759/cureus.9571

**Published:** 2020-08-05

**Authors:** Kashif A Hashmi, Hadi Y Saeed, Muhammad Shahzad Farid, Javeria Najam, Muhammad Irfan, Atif Ali Hashmi

**Affiliations:** 1 Cardiology, Chaudhry Pervaiz Elahi Institute of Cardiology, Multan, PAK; 2 Medicine, Liaquat National Hospital and Medical College, Karachi, PAK; 3 Statistics, Liaquat National Hospital and Medical College, Karachi, PAK; 4 Pathology, Liaquat National Hospital and Medical College, Karachi, PAK

**Keywords:** multivessel coronary artery disease (mvcad), st segment elevation myocardial infarction (stemi), non-st segment elevation myocardial infarction (nstemi), acute coronary syndrome (acs), troponin t, unstable angina, myocardial infarction (mi)

## Abstract

Introduction

Non-ST segment elevation myocardial infarction (NSTEMI) is becoming more common than ST segment elevation myocardial infarction (STEMI) and data regarding presence of underlying multivessel coronary artery disease (MVCAD) in these patients is consistent in locoregional population that leads to lethal delays in proper management. Therefore, in the current study, we aimed to evaluate the frequency of MVCAD in NSTEMI with markedly raised troponin T levels. This will help to identify patients that should be labeled as high risk and must be referred for coronary revascularization on priority basis, so that clinical outcomes can be improved in these patients.

Methods

This cross-sectional research study was carried out at Chaudhary Pervaiz Elahi Institute of Cardiology, Multan over a period of one year. A total of 326 patients with history of chest discomfort within past 48 hours of presentation or angina equivalent symptoms and cardiac troponin T more than 500 ng/l were included in the study. Coronary angiography was done within 72 hours of same hospital admission. The outcome variable i.e. MVCAD was determined.

Results

Mean age of patients was 50.74 ± 7.75 years with range of 30 to 60 years. MVCAD was found in 107 (32.82%) patients, whilst there was no MVCAD in 219 (67.18%) patients. Moreover, no significant association of MVCAD was noted with age or smoking.

Conclusion

We found presence of MVCAD in a considerable number of patients presenting with NSTEMI. The key to detect the underlying presence of MVCAD in these patients is lifted troponin T levels. Therefore, we conclude that any patient with elevated troponin T levels, even in the absence of ST segment elevation, should undergo cardiac catheterization to detect presence of MVCAD as this subset of patients can benefit from early revascularization including coronary artery bypass graft (CABG) surgery.

## Introduction

Myocardial infarction is the part of acute coronary syndrome (ACS) that includes unstable angina, non-ST-segment elevation myocardial infarction (NSTEMI) and ST-segment elevation myocardial infarction (STEMI). Coronary artery disease is the leading killer of individuals worldwide and kills over 6 million individuals each year. NSTEMI is becoming more common than STEMI with the passage of time. The annual incidence is 73 per 1000 inhabitants, but differs among different countries with average hospital mortality rate of 3-5% [1-3].

Although STEMI is considered more dangerous as it reflects full thickness infarction of myometrial wall, there is now convincing evidence that NSTEMI may harbor a severe underlying disease in a significant number of cases. Over recent days, American College of Cardiology (ACC) and National Cardiovascular Database Registry (NCDR) outlined that 42% of NSTEMI patients had multi-vessel coronary artery disease (MVCAD) that requires an aggressive management approach [4]. MVCAD is defined by the presence of ≥50% diameter stenosis of two or more epicardial coronary arteries. The presence of MVCAD indicates poorer prognosis and a significantly higher mortality than single-vessel disease [5]. The affected arteries can be reopened using an angioplasty or rerouted using cardiac bypass surgery. In addition, thrombolysis may also lead to deceased frequency of left ventricular thrombus formation [6,7].

It is postulated that events underlying ACS, i.e. disruption or rupture of atherosclerotic plaque, are due to an underlying inflammatory process [8]. Cardiac troponin T is a component of contractile apparatus of cardiomyocytes and is preferred biochemical marker of myocardial infarction and necrosis in patients with NSTEMI. The testing for troponins is stipulated a major indication for early risk stratification according to ACC/American Heart Association (AHA) Guidelines, as it ameliorates the clinical outcomes in NSTEMI patients [9]. The outcome in patients with ACS is proportionate to the levels of serum troponin levels [10].

Although troponin T is considered as a marker of myocardial wall damage, data regarding its ability to predict the presence of underlying MVCAD is inconsistent in the locoregional population, a few studies reported an extensive CAD with only borderline troponin elevation [11,12], on the other hand studies have also shown a high frequency of MVCAD with NSTEMI with high troponin levels. Therefore, in the current study, we aimed to evaluate the frequency of MVCAD in NSTEMI with markedly raised troponin T levels. This will help to identify patients that should be labeled as high risk and must be referred for coronary revascularization on priority basis, so that clinical outcomes can be improved in these patients.

## Materials and methods

This cross-sectional research study was carried out at Chaudhary Pervaiz Elahi Institute of Cardiology, Multan over a period of one year. A total of 326 patients were enrolled. Patients of either gender, aged 30 years to 60 years that presented with chest pain or heaviness or other symptoms related to angina in the past two days along with cardiac troponin T more than 500 ng/l (normal value <14 ng/l) were included in the study. Patients with electrocardiogram (ECG) changes indicative of STEMI along with those whose ECG showed left bundle branch block or pathological Q waves were excluded from the study. Patents with previous cardiac interventions or renal insufficiency (serum creatinine > 1.4 mg/dl) and patients who denied to undergo coronary angiography during hospitalization were also excluded from the study.

Complete history and examination was done. Blood samples for cardiac troponin T were drawn after 8 hours and 48 hours of hospital admission. Troponin T was determined using COBAS ELECTROSYS 2010 ROCHE instrument. The troponin T more than 500 ng/l was labeled as markedly raised. Coronary angiography was done within 72 hours of same hospital admission. Coronary angiographic films were analyzed by a senior cardiologist having five-year post fellowship experience and lesion was assessed according to standard protocols. The outcome variable, i.e. MVCAD along with demographic profile, was entered in a pre-designed proforma and recorded.

Statistical analysis

Data was scrutinized by operating on statistical package for social sciences (SPSS) version 21 (IBM Corp., Armonk, NY). Mean and standard deviation were evaluated for quantitative variables while frequency and percentage were calibrated for qualitative variables. Stratification was done with reference to qualitative variables to see the effect of these modifiers on multi-vessel severe coronary artery disease by using chi square test. p ≤ 0.05 were considered as significant.

## Results

Out of the 326 patients, 252 (77.30%) were males and 74 (22.70%) were females with ratio of 3.4:1. Mean age of patients was 50.74 ± 7.75 years with range of 30 to 60 years. Majority of the patients (179, 54.91%) were between 51 to 60 years of age. A total of 223 (68.4%) patients of study population were smokers while ECG changes were found in 192 (58.9%) patients. In our study, MVCAD was found in 107 (32.82%) patients, whilst there was no MVCAD in 219 (67.18%) patients. Detailed characteristics of study population are presented in Table 1.

**Table 1:** Characteristics of study population °Mean ± SD

Characteristic	n (%)
Gender	
Male	252 (77.3)
Female	74 (22.7)
Age (years)°	50.74 ± 7.75
Age Group	
30-40 years	33 (10.1)
41-50 years	114 (35)
51-60 years	179 (54.9)
Smoker	
Yes	223 (68.4)
No	103 (31.6)
ECG Changes	
Yes	192 (58.9)
No	134 (41.1)
Multi-vessel severe coronary artery disease	
Present	107 (32.8)
Absent	219 (67.2)

When stratification was done on age groups reciprocal to MVCAD, it was recorded that there was no remarkable difference of MVCAD among different age groups (p = 0.984) while gender stratification has also shown no statistically notable difference (p = 0.630). Similarly, no significant association of MVCAD was noted with smoking status (p = 0.476) and ECG changes (p = 0.807). Detailed results of association are presented in Table 2.

**Table 2:** Association of multi-vessel coronary artery disease with characteristics of study population Chi-Square test was applied. P-value ≤ 0.05, considered as significant.

	Multi-vessel severe coronary artery disease		P-value
	Present (n = 107)	Absent (n = 219)	
Gender			
Male	81 (75.7)	171 (78.1)	0.630
Female	26 (24.3)	48 (21.9)	
Age Group			
30-40 years	11 (10.3)	22 (10)	0.984
41-50 years	38 (35.5)	76 (34.7)	
51-60 years	58 (54.2)	121 (55.3)	
Smoker			
Yes	76 (71)	147 (67.1)	0.476
No	31 (29)	72 (32.9)	
ECG Changes			
Yes	62 (57.9)	130 (59.4)	0.807
No	45 (42.1)	89 (40.6)	

## Discussion

In the current study, we found out that a substantial number of patients with NSTEMI having markedly elevated troponin T levels had underlying MVCAD, that necessitated urgent revascularization including coronary artery bypass graft (CABG) surgery. Moreover, no significant association of MVCAD was noted with age or smoking.

According to severity, myocardial infarction can be branched into two types; NSTEMI is the less intrusive type. In NSTEMI, the blood clot partially blocks up the artery, and as a consequence only a section of the heart muscle being supplied by the influenced artery leads to ischemia. In contrary to the more intensified form of heart attack (STEMI), the NSTEMI does not account for attributable elevation in the "ST segment" portion of the ECG. Therefore, it signifies that in NSTEMI, the artery is not entirely occluded [10,13]. Considerable advances in the discernment of myocardial injury and necrosis have been made over the past few decades. Accordingly, the definition of MI has metamorphosed through the years.

A study demonstrated that patients with NSTEMI account for the majority (54%) of acute MI patients admitted to the hospital. This study also revealed that patients with NSTEMI had higher one-year mortality (31%) than patients with ST-elevation MI (21%). Patients with NSTEMI tend to be older, have poorer LV function, multi-vessel disease and history of acute coronary events [14].

Age range in this study was 30 to 60 years with mean age of 50.74 ± 7.75 years with bulk of the patients i.e. 179 (54.91%) were between 51 to 60 years of age. Compared to other regional studies our mean age was slightly lower that may correlate with the increasing trend of cardiovascular diseases in younger age groups [15]. Whether this is a true trend or just due to better investigative potential needs to be ascertained.

MVCAD was noted in 107 (32.82%) patients, however there was no MVCAD in 219 (67.18%) patients in our study. Altmann et al. in their study have shown the frequency of MVCAD in 54% patients with NSTEMI [16]. Another study by Qadir et al., which included 230 NSTEMI patients, found that in 111 patients with cardiac troponin T levels ≤10 folds upper limit of normal, 25 (22.52%) had single vessel, 40 (36%) had two vessel and 34 (30.6%) had three vessel significant CAD, whereas in 119 patients with cardiac troponin T levels >10 folds upper limit of normal, 23 (19.3%) had single vessel, 37 (31.1%) had two vessel and 55 (46.2%) had three vessel significant CAD [17]. Other studies reported frequency of MVCAD in NSTEMI ranging from 40 to 65% [18,19], while a large ACC National Cardiovascular Database Registry report that included over hundred thousand patients, MVCAD was noted to be present in 42% of patients with NSTEMI [4]. Compared to these studies, frequency of MVCAD in our studied population was found to be slightly lower. This may be attributable to some different population characteristics in our study.

There exists some limitations to this study. Firstly, long-term follow-up was not done to detect mortality rates over a period of time. Secondly, this is a single institution data which may not be representative of entire population.

## Conclusions

MVCAD was found to be present in a large number of patients that presented with NSTEMI along with elevated troponin levels in our study. Therefore, we recommend that every patient with elevated troponin levels despite absence of ST segment elevation in ECG should have cardiac catheterization to evaluate the presence of underlying MVCAD. Early detection of MVCAD in these patients may lead to improved patient outcomes.
